# One-stage tubularized urethroplasty using the free inner plate of the foreskin in the treatment of proximal hypospadias

**DOI:** 10.1186/s12887-022-03464-2

**Published:** 2022-07-05

**Authors:** Tong Shi, Yan-Kun Lin, Qiao Bao, Wei-Hua Lao, Ke-Yu Ouyang

**Affiliations:** Pediatric Urology, Guangdong Maternal and Child Health Hospital, No. 521, Xingnan Avenue, Panyu District, Guangzhou, 511400 China

**Keywords:** Free inner plate of prepuce, Tubularized urethroplasty, Proximal hypospadias, Short-term efficacy

## Abstract

**Objective:**

This study summarizes the short-term efficacy of the one-stage tubularized urethroplasty using the free inner in proximal hypospadias.

**Methods:**

A retrospective analysis was conducted on 42 patients with proximal hypospadias. All cases were treated with one-stage tubularized urethroplasty from January 2020 to June 2021. The postoperative complications like urethral fistula, urethral stricture, diverticulum, and split penis head were recorded.

**Results:**

Patients were followed up for 3 to 15 months (an average of 8.5 months). A total of 26 cases (62%) were repaired without any complication. Five patients (11.9%) developed urinary fistulas and underwent secondary repair: three cases with anastomotic fistulas and two cases of coronal fistulas. Nine patients (21.4%) had stenosis of the head segment of the penis, six (14.3%) had stenosis that was relieved by urethral dilatation combined with topical mometasone furoate 1 month after urethral catheter removal. Two patients (4.8%) had severe stenosis with secondary surgical stenosis incision, and one (2.4%) had combined urethral diverticulum in which urethral stenosis incision and diverticulectomy were performed.

**Conclusions:**

Tubularized urethroplasty using the free inner bears the advantages of easy access, reduced short-term complications, low incidence of diverticula.

## Introduction

Hypospadias is a common congenital defect of the genitourinary system in children. The incidence of hypospadias is approximately 18.6 in infant boys in Europe. Its incidence is the highest in North America at 34.2 per 10,000 births (ranging from 6 to 129.8) and the lowest in Asia at 0.6% per 10,000 births [1]. The pathogenic factors of this disease remain complex. Mounting studies have shown the lack of clarity regarding a definite etiology for hypospadias, with some children suspected to be induced by a single gene mutation [2, 3]. Hypospadias is associated with low birth weight, low endocrine levels, anti-epileptic drugs, ovulation-promoting drugs, and advanced maternal age [4]. Clinically, the affected patients are primarily treated surgically. However, there are different methods and types of surgery, with a varying scope of application, surgical outcome, and prognosis [5]. Therefore, in clinical practice, the surgical approach should be chosen appropriately to achieve better outcomes, fewer complications, and improved prognosis [6]. Controversy prevails in national and international research regarding the performance of a one-stage or two-stage urethroplasty for proximal hypospadias. Springer et al. claimed that specialists in proximal hypospadias repair were consistent in their preference for one-or two-stage surgery, while younger surgeons had a strong predilection for staged urethroplasty in patients [6, 7]. None of the currently available procedures display a high level of patient satisfaction. Duckett’s procedure is considered one of the main surgeries for severe hypospadias with a long urethral defect. However, such a method generally exhibits a high incidence of postoperative urethral diverticula due to the presence of a bloated and rotated penis after forming a vascular pedicle. One study reported that over 50% of patients treated for proximal and complex hypospadias were dissatisfied with the appearance of their penis [8].

Devine and Horton first reported the use of free foreskin grafts in hypospadias surgery [9, 10]. Few studies have been reported on free foreskin grafts. The current retrospective analysis was conducted on 42 patients with proximal hypospadias. They underwent one-stage tubularized urethroplasty using the free inner plate of the foreskin by the same surgeon from January 2020 to June 2021. All cases were treated with one-stage tubularized urethroplasty with the free inner plate of the prepuce. The bending degree of the penis, the length of the urethral defect after correction of penile bending, and postoperative complications like the appearance of urethral fistula, urethral stricture, diverticulum, and split penis head were recorded. The report is as follows.

## Material and methods

### General data

A retrospective analysis was conducted on 42 children aged 18 months to 6 years old with a mean age of (40.5 ± 21.4) months old. The children with severe hypospadias were admitted from January 2020 to June 2021 and underwent one-stage urethroplasty using the free inner plate of the prepuce. All procedures were performed by the same experienced surgeon. The median follow-up time was 8 months (1–16 months). Eligible patients were followed up regularly in the clinic via WeChat, video calls, and outpatient visits 2 weeks, 3 months, 6 months, and 1 year after the surgery. The 42 patients included five perineal cases (12%), 11 scrotal cases (26%), and 26 penile root cases (62%).

### Operation method

**Fig. 1:**
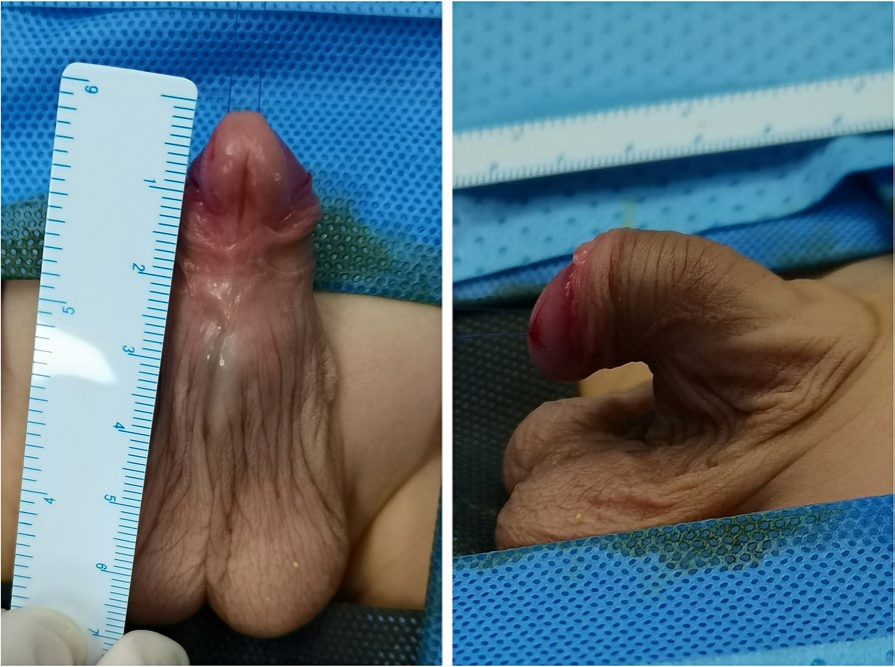
Preoperative appearance

**Fig. 2:**
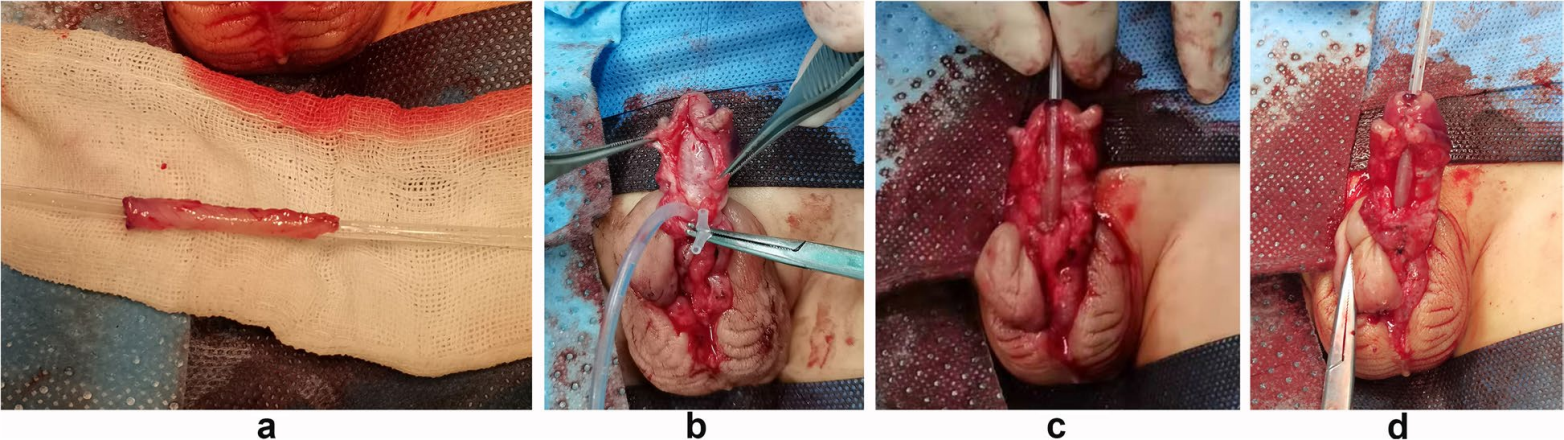
**a** The free skin flap. **b** loosened and corrected the bowstring relationship between the urethral plate and the corpus cavernosum. **c** the distal part of the skin tube and the penis head were sutured to form the urethral orifice. **d** The urethra was covered with bush fascia and subcutaneous tissue

**Fig. 3:**
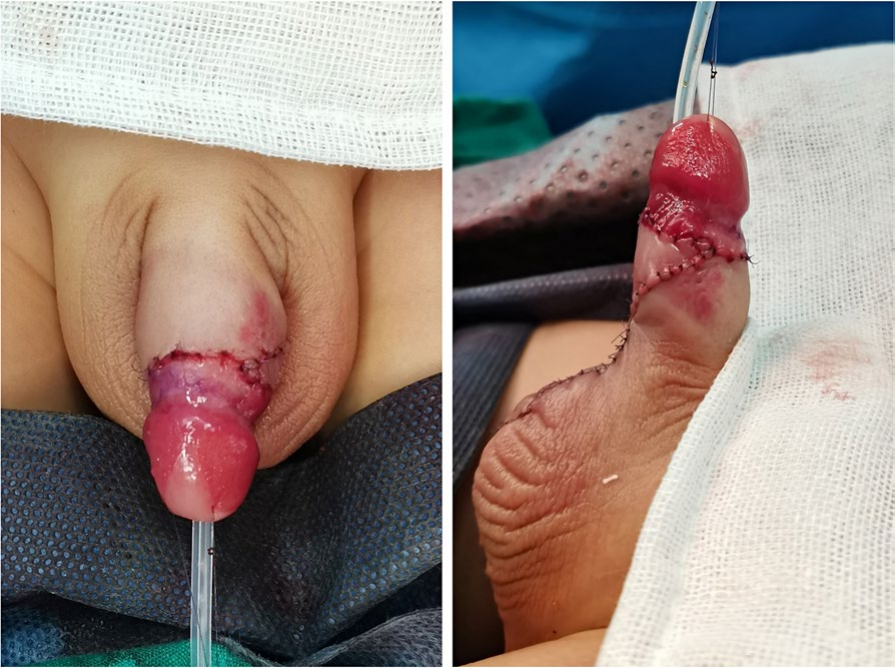
Postoperative appearance

The technique of tubularized urethroplasty using the free inner plate of the foreskin was adopted. The penis head was pulled (Fig. 1), a circular incision was made 0.6–0.7 cm below the coronal sulcus, and the urethral plate was transected. A U-shaped skin flap was made along both sides of the cavernous body of the urethra, and the ventral thin part of the urethra was cut to reach the bifurcation position of the urethral cavernous body. The scrotum was cut longitudinally at the proximal midline of the urethral orifice. The penis was detached, and the ventral dense fibers were completely loosened to reach the bulbar part of the urethra. The urethral plate was transected and dissected deeply along the superficial white membrane of the corpus cavernosum; this fully loosened and corrected the bowstring relationship between the urethral plate and the corpus cavernosum. Saline was injected into the corpus cavernosum, and an artificial erection test was performed to check the correction of the curved urethral penis. The residual urethral plate was cut, and the marginal unhealthy tissues were removed. The traction thread was sewed on the dorsal prepuce inner plate. The width was determined by the distance between the distal part of the penis head and the urethral stump, and the length was determined as twice the circumference of the urinary catheter; the skin was cut by transverse rectangular skin tangent. The subcutaneous connective tissue was separated from the edge of the skin flap, and the skin in the edge area was fully extended. A 10F urine tube was used to wrap the free skin flap (Fig. 2), a 7–0 absorbable thread was used to sew the wound tube continuously and then intermittently to form a free skin tube, and the two ends of the skin tube were trimmed with oval slopes (to reduce anastomotic stenosis and urethral stricture). A 7–0 absorbable thread was used to stitch the stump of the urethral plate intermittently to the anastomosis. The penis was dissected in a wing-shaped line, and the redundant tissues of the corpus cavernosum on both sides of the penis were deeply trimmed to reduce the volume of the penis. A 7–0 absorbable thread was used to sew the corpus cavernosum on the penis head to wrap the distal section of the skin tube to form the penis head. Using a 7–0 absorbable thread, the distal part of the skin tube and the penis head were sutured to form the urethral orifice. The urethra was covered with bush fascia and subcutaneous tissue, and the anastomosis was covered with scrotal fascia; this was followed by trimming and suturing of the foreskin (Fig. 3). The gauze and elastic adhesive tape were used to press and fix the perineum. Three days after the operation, the outer layer was not pressurized, and overflow, infection, necrosis, and bleeding were observed. The membrane was removed and the dressing was changed. If no obvious abnormality was found, the membrane was removed seven days after the operation, and the wound was washed with 0.1% Anduofu. The urinary catheter was removed two to three weeks after the operation.

## Results

The hypospadias included five perineal types (12%), 11 scrotal types (26%), and 26 penile root types (62%) with a median length of the free-formed urethra of 35 mm (range: 30–60 mm, mean value: 38 mm).

A total of 26 cases (62%) were repaired in the one-stage surgery without any complication. Five patients (11.9%) developed urinary fistulas and underwent secondary repair, including three cases with anastomotic fistulas and two cases of coronal fistulas. Nine patients (21.4%) had stenosis of the head segment of the penis, six (14.3%) had stenosis that was relieved by urethral dilatation combined with topical mometasone furoate 1 month after urethral catheter removal. Two patients (4.8%) had severe stenosis with secondary surgical stenosis incision, and one (2.4%) had combined urethral diverticulum in which urethral stenosis incision and diverticulectomy were performed. No residual recurved penis and penis head split was observed after the operation.

## Discussion

Severe hypospadias is a common pediatric urological condition that is accompanied by urethral dysplasia and may result in severe chordee [11]. The urethral plate is severed to straighten the penis in affected children. This causes a long segment of the urethra to be defective and requires repair and reconstruction [6]. Such a surgical method remains controversial up to a point owing to the difficulty of the operation and the postoperative complications. Currently, some variations can be noted in the clinical approach toward treating the disease at the national and international levels. The hypospadias surgery ensures that the penis is essentially normal in appearance and can be erected and straightened normally while ensuring proper urinary flow and a normal urethral opening [12]. Most scholars mainly adopt a staged surgical treatment [13]. However, in the one-stage repair, the urethroplasty using the transverse penile island flap (Duckett method) is preferred [14]. To maximize the effectiveness of the treatment and reduce complications, it is necessary to improve continuously the technical proficiency of performing one-stage repair of hypospadias.

In the treatment of severe hypospadias, the first step is to transect the urethral plate to correct the downward curvature of the penis as much as possible, regardless of the surgical option [15]. During the urethroplasty, the risk of surgical failure and postoperative complications is greatly increased if the newly formed urethra fails to secure the blood supply [16]. The tubularized urethroplasty using the free inner plate of the foreskin is a novel surgical procedure that has been used in several centers since Devine and Horton first reported its application in the hypospadias. However, results to date have varied in different reports and are less well reported. Some researchers have even concluded that free foreskin grafts are not suitable for hypospadias surgery due to their high rate of complications [17]. In contrast, a recent article reported that one-stage tubularized urethroplasty using the free inner plate of foreskin achieved good functional and cosmetic results with a low rate of postoperative complications [18]. This offers greater advantages compared to conventional surgery.

There were five perineal types (12%), 11 scrotal types (26%), and 26 penile root types (62%) with a median length of the free-formed urethra of 35 mm (range: 30–60 mm, mean value: 38 mm). The risk and difficulty in performing the tubularized urethroplasty lie in the reconstruction of the blood supply. During the operation, it was necessary to ensure that the skin was thinly incised and the subcutaneous tissue was removed as much as possible when the inner plate of the prepuce was dissociated. This precaution ensured a quick absorption of nutrition from the wound surface of the surrounding tissue. The width of the free foreskin was about 1.2–1.5 cm, about 2 times that of the urinary catheter. It was essential to ascertain that the type of the urinary catheter matched the width of the free foreskin. The use of a very small catheter may result in insufficient pressure, and the skin patch and the receiving area may not combine firmly to form a dead space. Moreover, it also avoids the situation of nutrition deficiency and necrosis caused by the obstruction of the growth of new blood vessels into the skin. In addition, as shown in Fig. 2 b, the fibrous connective tissue on the ventral side of the corpus cavernosum was completely separated and removed to ensure sufficient blood supply to the fascia tissue. Proper pressurization ensured that the free skin tube and the ventral side of the penis were well attached and the blood supply to the fascia tissue is not obstructed.

**Fig. 4:**
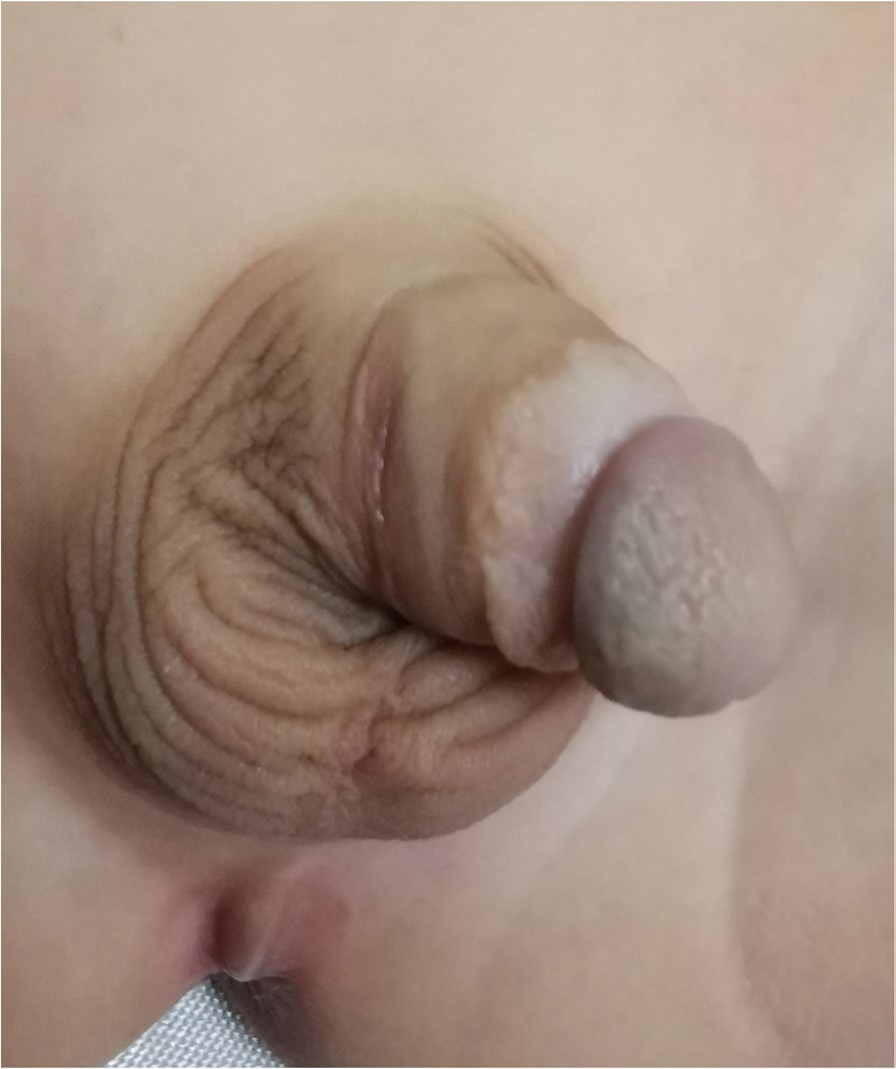
Appearance 6 months after surgery

As shown in Fig. 2 a, in the urethral reconstruction with the free inner plate of the prepuce, the diameter of the skin tube was the same, and both ends were oval. This guaranteed the uniform extension of the epithelium and avoided the skin tube formed by the pedicled skin flap being too wide. It easily formed the diverticulum and avoided the formation of the skin fold due to the limitation of the pedicled tissue. This method improved the function and cosmetic effect (Fig. 4), for it eliminated the secondary torsion caused by the vascular pedicle and enlargement of the penis trunk [19]. This was particularly evident in patients with smaller penises. When anastomosing with the original urethral orifice and new urethroplasty, attention should be paid to the oval shape of the anastomotic stoma, which can reduce the occurrence of anastomotic stenosis. No cases of anastomotic stenosis were found in this study. Thus, anastomotic stoma stenosis was avoided by this method. One case noted in this group presented a secondary urethral diverticulum with a thin urinary line, poor urethral dilatation, and significant urethral stricture in the head segment of the penis. The head segment of the penis was covered by direct sutures as there was no subcutaneous tissue covering the urethra. Whether the cause of stenosis of the head segment of the penis was related to the inadmissibility of the penis head and the technique of phalloplasty needs to be further explored. In this group, 26 cases (62%) were repaired in one stage without any complications. Five patients (11.9%) developed a urinary fistula and received a secondary repair, below the accepted level of urethral fistula. It was crucial to preserve the well-vascularized tissues on both sides of the urethral plate that covered the urethra for protection when the penis and foreskin were decapsulated. The caliber of the new urethra can be further optimized with the support of the urethra and the reconstruction of the blood supply to the outer fascia. The wide fascia remaining after freeing the foreskin was well preserved and transferred ventrally via the sides of the penis, ensuring a loose and homogeneous circulatory support of the inner plate skin tube. The free skin tube and the urethral plate were anastomosed in a more flexible way for capping. The postoperative visit informed us that 90% of children's families were satisfied with the appearance but were less satisfied with dysuria and urethral diverticulum. During the operation, the transection of the urethral plate and urethroplasty were long enough, and no residual recurved penis was found in this study.

The limitations of our research included data obtained from a single institution, the relatively small sample size, and the lack of objective assessment of urination function. Due to the short follow-up period and the fact that the children had not reached puberty, a post-pubertal assessment, including sexual function could not be performed. A multicenter study with larger sample size is needed to confirm the effectiveness of the tubularized urethroplasty. Mounting scholars supported that tubularized urethroplasty using the free inner plate of the foreskin must be abandoned due to the high rate of complications, particularly in patients with moderate to severe hypospadias. In contrast, our results suggested that tubularized urethroplasty using the free inner plate of the foreskin was an appropriate option for the repair of hypospadias. Our procedure achieved good functional and cosmetic results, with a relatively low short-termed postoperative morbidity. Moreover, it reduced the pain and family burden for secondary surgery in most children with successful one-stage operation without complications. Addtionally, the small sample size and the short follow-up time may influence the results. Therefore, in the next work, we plan to carry out more studies with large sample sizes and prolong follow-up after surgery to investigate on the long-term complications of one-stage tubularized urethroplasty.

## Data Availability

All data generated or analyzed during this study are included in this article. Further inquiries can be directed to the corresponding author.
